# Gambling in COVID-19 Lockdown in the UK: Depression, Stress, and Anxiety

**DOI:** 10.3389/fpsyt.2021.621497

**Published:** 2021-01-25

**Authors:** Steve Sharman, Amanda Roberts, Henrietta Bowden-Jones, John Strang

**Affiliations:** ^1^National Addiction Centre, Institute of Psychiatry, Psychology and Neuroscience, Kings College London, London, United Kingdom; ^2^School of Psychology, University of East London, London, United Kingdom; ^3^School of Psychology, University of Lincoln, Lincoln, United Kingdom; ^4^National Problem Gambling Clinic, London, United Kingdom; ^5^Department of Psychiatry, University of Cambridge, Cambridge, United Kingdom; ^6^Faculty of Brain Sciences, University College London, London, United Kingdom

**Keywords:** gambling, COVID-19, depression, stress, anxiety, disordered gambling

## Abstract

To combat the spread of COVID-19, the UK Government implemented a range of “lockdown” measures. Lockdown has necessarily changed the gambling habits of gamblers in the UK, and the impact of these measures on the mental health of gamblers is unknown. To understand the impact of lockdown on gamblers, in April 2020, after ~6 weeks of lockdown, participants (*N* = 1,028, 72% female) completed an online questionnaire. Gambling engagement data was collected for pre-lockdown via the Brief Problem Gambling Screen (BPGS) allowing participants to be classified as Non-Gamblers (NG), Non-Problem Gamblers (NPG) or Potential Problem Gamblers (PPG). The Depression, Stress, and Anxiety Scale (DASS21) was used to measure depression, stress, and anxiety scores both pre- and during-lockdown. Results indicate that depression, stress and anxiety has increased across the whole sample. Participants classified in the PPG group reported higher scores on each sub scale at both baseline and during lockdown. Increases were observed on each DASS21 subscale, for each gambler group, however despite variable significance and effect sizes, the magnitude of increases did not differ between groups. Lockdown has had a significant impact on mental health of participants; whilst depression stress and anxiety remain highest in potential problem gamblers, pre-lockdown gambler status did not affect changes in DASS21 scores.

## Introduction

The global COVID-19 pandemic has had a significant impact on the lives of people around the world. In the UK, government measures implemented to stop the spread of the virus resulted in much of society being in “lockdown” from late March, with measures only being eased in late June and early July. Lockdown impacted on individuals, families, and wider society from different perspectives; interestingly, some of these impacts may have led to changes in addictive behaviors due to reduced accessibility of substances, withdrawal, increased craving, removal of positive reinforcers, and reduced access to medical or psychological support (1).

Gamblers were potentially at greater risk of gambling-related harm (2), as lockdown potentially exacerbated established risk factors for disordered gambling, including social isolation (3–5), lack of social support (6), boredom, (7, 8), and financial insecurity (9–11).

Furthermore, depression, stress, and anxiety disorders are common in gamblers; elevated levels of depression and anxiety are frequently observed in treatment-seeking disordered gamblers (12–15). A meta-analysis and systematic review of co-morbid mental health disorders in treatment seeking gamblers identified 36 studies, and reported that 23.1% of gamblers presented with a current mood disorder, 17.6% with an anxiety disorder, and 29.9% with a major depressive disorder (16). Further studies have found that severity of gambling problems was significantly associated with severity of depressive symptoms (17, 18). Within those who gamble, problem gamblers scored more highly on depression and anxiety scores than non-problem gamblers (19). Additionally, depressive symptoms are also more common in those who gamble when recruiting from population samples. In a systematic review, Lorains et al. (20) identified 11 studies that recruited from general populations and reported an average effect size of 23.2% for major depression, 37.4% for any anxiety disorder, and 11.1% for generalized anxiety disorder.

Whilst co-morbidities between gambling, depression and anxiety are well-evidenced, the direction of the effect is less clear. Depression can precede gambling, with gambling used to escape from or relieve negative emotions, however the converse is also true; gambling can lead to financial and social difficulties, that in turn lead to depression (21). Similarly, stress has also been identified as both a reason to gamble (22, 23), and a consequence of gambling (24, 25), whilst altered stress physiology can render an individual predisposed to development of gambling disorder (26, 27). For a comprehensive overview of gambling and stress, see Buchanan et al. (28).

The unprecedented nature of lockdown in the UK means the short- and longer-term impacts of lockdown on depression, anxiety and stress in gamblers are unknown. This study aims to provide the first analysis of mental health change in gamblers, as a function of pre-lockdown gambling disorder severity.

Specially, the study has the following aims:

- To measure whether lockdown has affected depression, stress and anxiety.
- To understand if lockdown has affected depression, stress and anxiety as a function of gambler risk category.

## Materials and Methods

Even prior to the enduring research climate which has restricted face-to-face social interaction, remote data collection had become more frequently utilized in social science research (29), and has previously been used for gambling research (30, 31). Online participant pools offer reliable, large-scale recruitment allowing rapid recruitment to studies (32). The present study was programmed in Qualtrics (https://www.qualtrics.com) and was then shared to the online participant recruitment pool, Prolific Academic. Registered Prolific users were then able to respond to the study advert, and assuming eligibility, complete the study. Prolific Academic was chosen over other crowd-sourcing platforms as participants recruited from Prolific Academic have been found to be more naïve and less dishonest than those recruited from alternative platform Amazon's Mechanical Turk (MTurk), and to produce higher quality data than alternative crowd-sourcing platform CrowdFlower (33).

All data were collected in a single online session. Data were collected across a week-long time window at the end of April 2020. In the single session, questions asked about behaviors covering two distinct time periods; the first time-period refers to a specified period prior to the government recommended social distancing measures and is henceforth referred to as pre-lockdown. Questions also asked participants to self-report behavior since being asked to socially isolate, referred to henceforth as during-lockdown.

### Participants

Participants were recruited through Prolific Academic. To maximize responses, the only eligibility criteria specified was that participants were required to be a current UK resident, and were adhering to some measure of social distancing, therefore were affected by lockdown. Thirteen participants were excluded as they were not engaged in any form of social distancing, resulting in a final sample of 1,028 participants (72.1% female; age *M* = 33.19, SD = 11.66, range 18–73). Age did not differ significantly between males (*M* = 32.68, SD = 12.26) and females (*M* = 33.46, SD = 11.45) [*t*_(990)_ = 0.94, *p* = 0.35]. All participants included in analyses were engaged in some level of measures to prevent the spread of COVID-19, either social distancing, social isolation, or social shielding. For convenience, the term social distancing is used henceforth to include all levels distancing measures. Participants were most commonly social distancing in a household with 2–3 other people (40.5%), and least commonly distancing alone (15%). Most were distancing with family (76.46%); 76.17% had been distancing for between 2 and 4 weeks, and 64.1% were employed, at the time of survey completion.

### Measures

Participants completed the short form of the Depression, Anxiety, and Stress Scale [DASS 21, (34)]. The DASS 21 is a self-completion measure that is comprised of 3 scales, each measuring a different dimension. Each scale has seven items measuring depression (dysphoric mood states), anxiety (arousal states), and stress (negative affectivity). Construct validity of the DASS 21 has been tested in a UK non-clinical sample, with a quadripartite model returning optimal fit (RCFI = 0.94), when considering three distinct subscales and overall factor of general psychological distress (35).

Problem gambling status was measured using the Brief Problem Gambling Screen [BPGS-5, (36)]. The BPGS consists of five yes/no binary questions, and was used due to its brevity, and robust psychometric properties. Model development indicated that five item model demonstrated high specificity (99.9%) and sensitivity (90.8%), and greater clarification accuracy than other two, three or four item models (36). A score of 1 or more indicates problem gambling, and a need for further assessment (37). The BPGS was used to group participants into non-gambler, non-problem gambler and potential problem gambler groups for subsequent analysis.

### Procedure

Data were collected in April 2020. Participants were invited to partake in the study through having a registered Prolific Academic account. Participants gave online consent, and were paid £6.28 p/h, pro-rata for estimated study completion time, resulting in a payment of £1.78 per participant, considered “fair” by Prolific Academic. After providing consent, participants completed basic demographic questions, before completing the DASS-21 and the BPGS. Participants also completed questions regarding COVID-19 symptoms and gambling behavior, reported elsewhere. The study protocol was approved by the School of Psychology Research Committee at the University of Lincoln, ref: 2020-2392, and the University of East London University Research Ethics Committee, ref: ETH1920-0207.

### Data Analysis

Raw scores on the DASS21 were analyzed between groups using repeated measures ANOVA models. Positively skewed data were SQRT(+1) transformed prior to statistical comparison. Where transformations did not correct skewness, equivalent non-parametric tests were used. A standard alpha of 0.05 was used, however Bonferroni adjusted alpha values were adopted to correct for multiple comparisons, where appropriate. To report the magnitude of differences between groups, eta squared was reported as a measure of effect size. Effect sizes were reported as either small (η^2^ = 0.01), medium (η^2^ = 0.06), or large (η^2^ = 0.14), (38). Change scores for DASS scales were calculated and compared using ANOVA models across gambling behavior change categories. Error bars represent the standard error mean [SD/sqrt (N)]. Sample distribution across depression, anxiety, and stress severity categories from the DASS were analyzed between pre- and during-lockdown using chi-squared models. Analyses of adjusted z score residuals identified *post-hoc* differences in chi-squared models using appropriately adjusted *p* values (39). For sub-group analyses, participants were grouped in to Non-Gamblers (NG, *n* = 523), Non-Problem Gamblers, as defined by indicating past-year gambling but scoring zero on the BPGS (NPG, *n* = 362) or Potential Problem Gamblers, as defined by scoring > 0 on the BPGS (PPG, *n* = 143).

## Results

### Whole Sample

DASS scales showed significant increases between pre-lockdown and during-lockdown for depression, anxiety, and stress (Table 1). For depression, chi-squared analysis indicated that risk category distribution across the three DASS subscales in the whole sample was significantly different between the two time periods [$\chi_{\left(4\right)}^{2}$ = 36.3, *p* < 0.001]. Analysis of adjusted z score residuals indicates significant decreases in the “normal” category (*p* < 0.001) and increases in the “extremely severe” category (*p* < 0.001). The omnibus model for anxiety was significant [$\chi_{\left(4\right)}^{2}$ = 12.79, *p* = 0.012]; *post hoc* tests did not indicate any category change distribution change significant at the adjusted alpha of 0.005, although the increase in “extremely severe” was significant at 0.05. The omnibus model for stress was significant [$\chi_{\left(4\right)}^{2}$ = 52.18, *p* < 0.001]; *post hoc* tests indicate a significant increase in the “extremely severe” category (*p* < 0.001).

**Table 1:** DASS scale scores, whole sample.

**DASS scale**	**Pre-lockdown**		**During-lockdown**		**Test statistics**		
	***M***	**SD**	***M***	**SD**	***t***	**(df)**	***p***
Depression	2.18	1.05	2.43	1.15	9.47	1027	<0.001
Anxiety	1.77	0.97	1.84	1.09	2.7	1027	0.007
Stress	2.45	0.86	2.55	1.04	4.39	1027	<0.001

### Non-gamblers, Non-problem Gamblers, and Potential Problem Gamblers

When analyzing between gambler groups, DASS scale scores reported for pre- and during-lockdown were compared between groups. Data were analyzed in repeated measures ANOVAs with factors of Time (pre- and during-lockdown), and Group (NG, NPG, PPG).

### Depression

**Figure 1:**
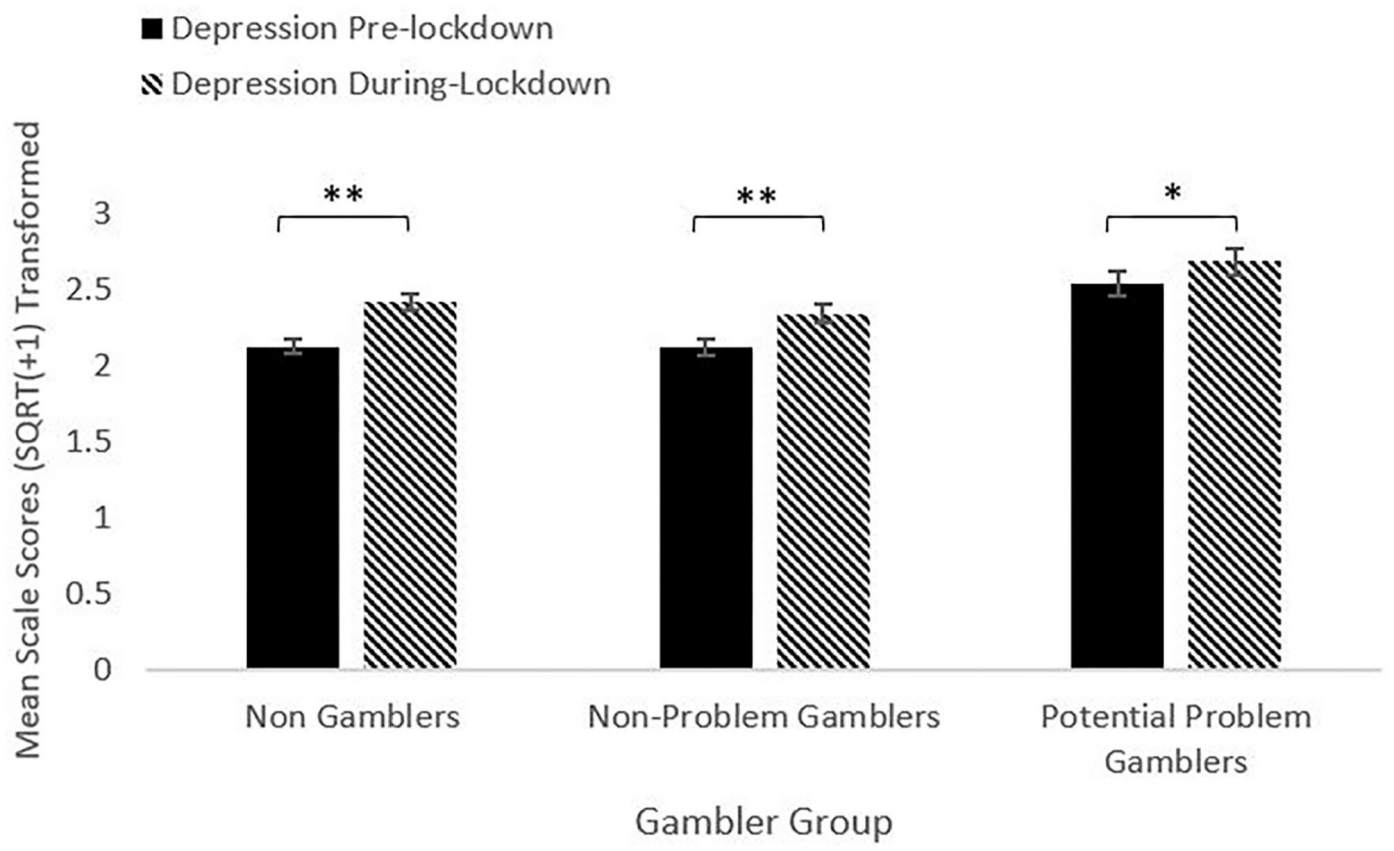
Depression pre- and during-lockdown by gambler group (**p < 0.001, *p < 0.05).

For depression, the repeated measures ANOVA model showed a significant main effect of Time [*F*_(1, 1025)_ = 55.83, *p* < 0.001, η^2^ = 0.052]. Using a Bonferroni corrected alpha of 0.016, the NG and NPG groups reported significant increases in depression between pre- and during-lockdown (lowest *t* = 5.13, *p* < 0.001). The PPG group reported an increase significant at 0.05, but not at the adjusted alpha [*t*_(142)_ = 2.28, *p* = 0.024], Figure 1.

The factor Group was also significant [*F*_(2, 1025)_ = 7.93, *p* < 0.001, η^2^ = 0.015]. The PPG group reported higher depression scores than both the NG and NPG groups (lowest *t* = 2.5, highest *p* = 0.013) for both pre- and during-lockdown. The NG and NPG groups did not differ from each other at either timepoint. The Time^*^Group interaction was not significant [*F*_(2, 1025)_ = 2.3, *p* = 0.10, η^2^ = 0.004]. The mean change score was calculated by subtracting scale score for pre-lockdown from the scale score for during-lockdown. Using a corrected alpha of 0.016, depression change scores did not significantly vary between any groups (highest *t* = 1.79, lowest *p* = 0.07).

### Anxiety

**Figure 2:**
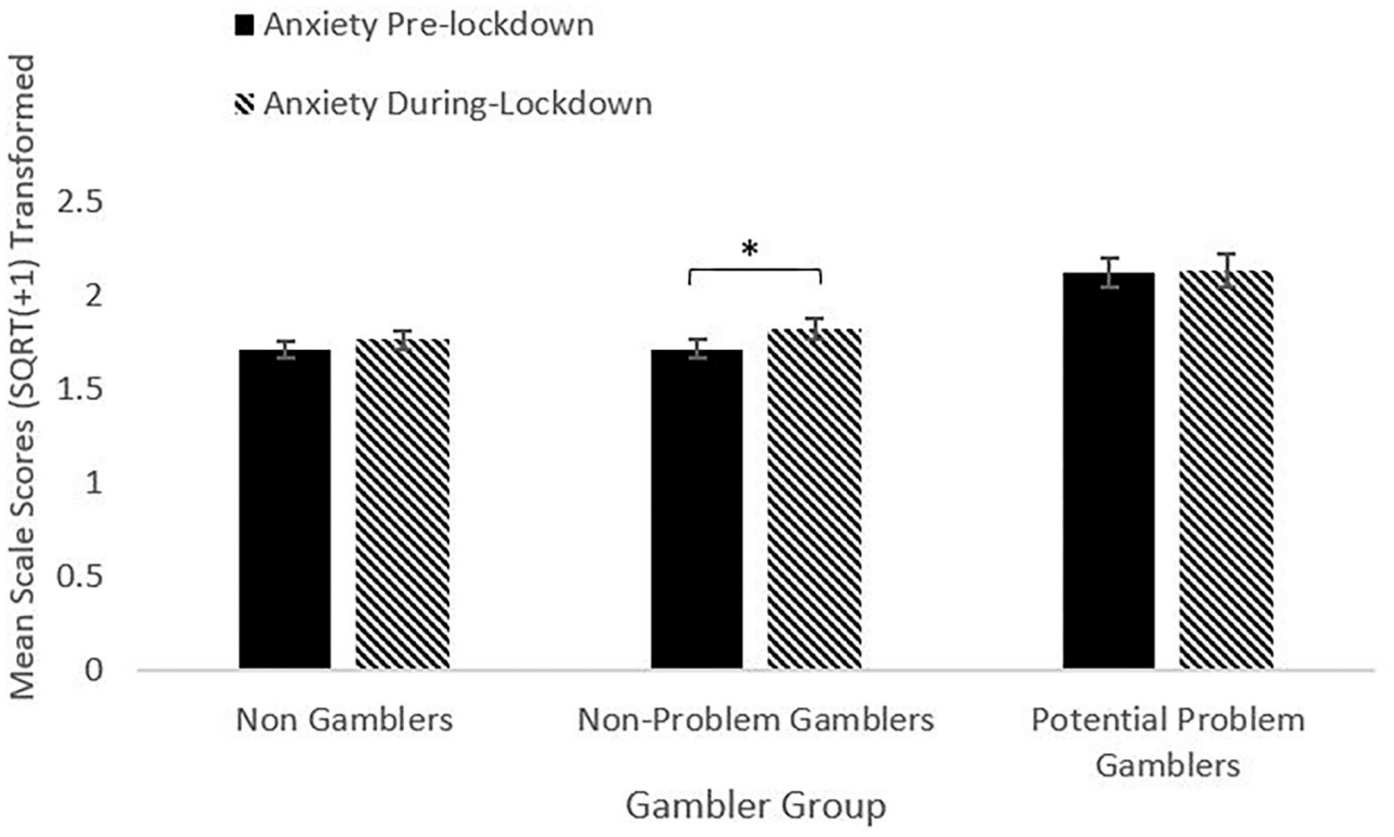
Anxiety pre- and during-lockdown by gambler group (*p < 0.05).

For anxiety, the repeated measures ANOVA model showed a significant main effect of Time [*F*_(1, 1025)_ = 3.95, *p* = 0.047, η^2^ = 0.004]. All groups reported an increase in anxiety between pre- and during-lockdown. The increase was significant for the NPG group [*t*_(361)_ = 2.64, *p* = 0.009], but not the NG or PPG groups (lowest *t* = 0.11, *p* = 0.91), Figure 2.

The factor of Group was significant [*F*_(2, 1025)_ = 9.74, *p* < 0.001, η^2^ = 0.019]. The PPG group reported significantly higher anxiety scores than the NPG and NG groups (lowest *t* = 3.03, highest *p* = 0.003) for both pre- and during-lockdown. The NG and NPG groups did not differ at either timepoint. The Time^*^Group interaction was not significant [*F*_(2, 1025)_ = 0.89, *p* = 0.411, η^2^ = 0.002]. The mean change score for anxiety did not differ between groups (highest *t* = 1.91, lowest *p* = 0.057).

### Stress

**Figure 3:**
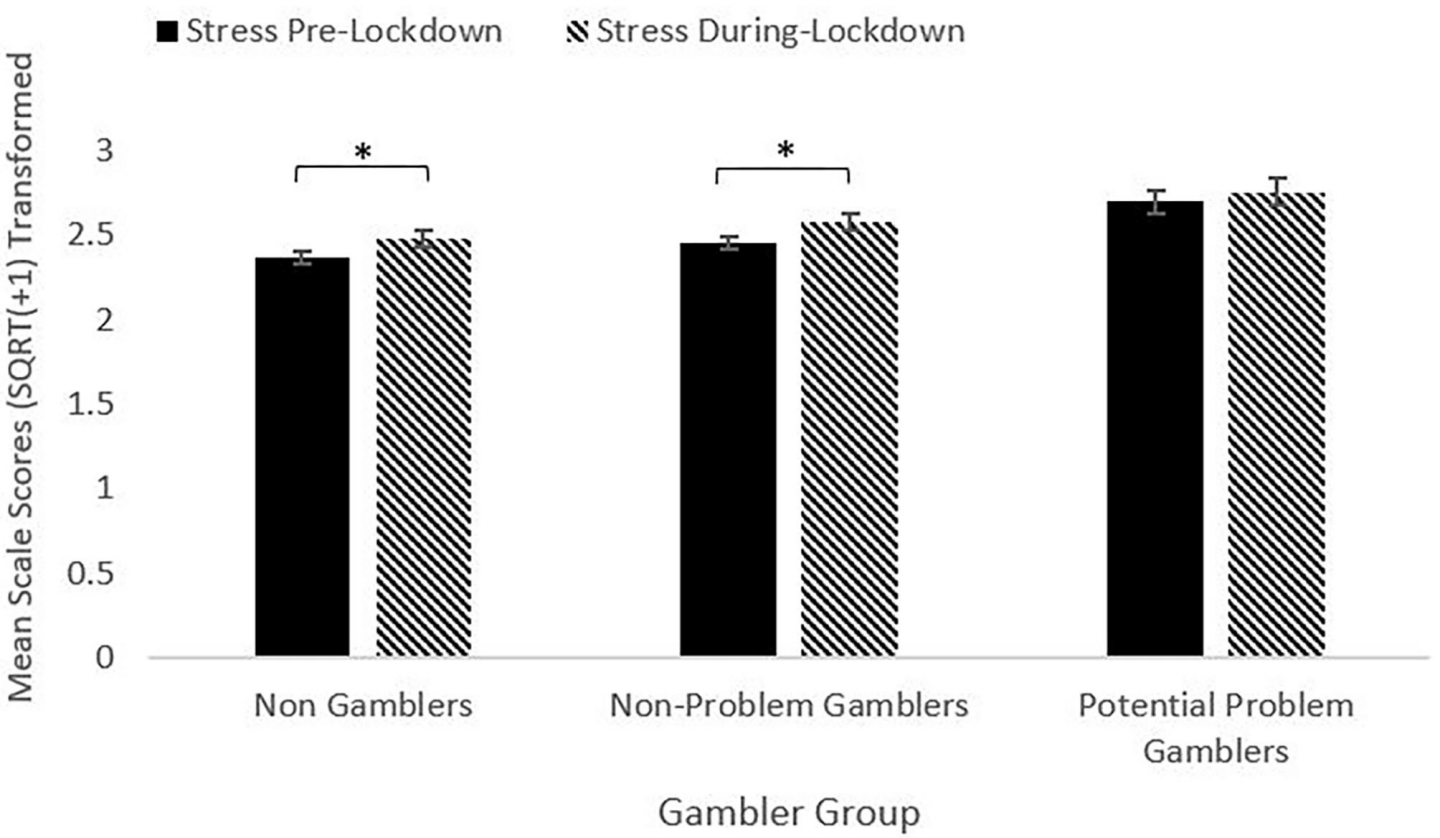
Stress pre- and during-lockdown by gambler group (*p < 0.05).

For stress, the repeated measures ANOVA model showed a significant main effect of Time [*F*_(1, 1025)_ = 11.89, *p* < 0.001, η^2^ = 0.011]. All groups reported an increase in stress between pre- and during-lockdown. The increase was significant for the NG and NPG groups (lowest *t* = 3.03, highest *p* = 0.003), but not for the PPG group [*t*_(142)_ = 0.91, *p* = 0.37], Figure 3.

The main effect of Group was also significant [*F*_(2, 1025)_ = 6.97, *p* = 0.001, η^2^ = 0.013]. The PPG group reported higher stress scores than the NG group at both periods (lowest *t* = 2.76, highest *p* = 0.006). The PPG group reported higher stress scores than the NPG group pre-lockdown [*t*_(503)_ = 3.19, *p* = 0.002], but not for during-lockdown [*t*_(503)_ = 1.88, *p* = 0.061]. The NG and NPG group did not differ at either time period. The Time^*^Group interaction was not significant [*F*_(2, 1025)_ = 0.36, *p* = 0.70, η^2^ = 0.001]. The mean change score for stress did not differ between groups at the adjusted alpha level, although change scores between the NG and PPG groups [*t*_(664)_ = 2.38, *p* = 0.018] and the NPG and PPG groups [*t*_(503)_ = 2.09, *p* = 0.038] were significant at 0.05.

## Discussion

The current study sought to provide some initial data on the influence of government enforced social isolation in response to the COVID-19 pandemic on depression, stress and anxiety in gamblers and non-gamblers in the UK. Recruiting a UK based online sample, preliminary results indicate the across the whole sample, levels of depression, anxiety, and stress have increased in lockdown, and that those who were classified as Potential Problem Gamblers reported, in general, higher levels of depression, stress, and anxiety.

### Depression

Across the whole sample, reported levels of depression increased significantly between pre- and during-lockdown. Within gambler groups, both the Non-Gambler (NG) and Non-Problem Gambler groups (NPG) reported significant increases in depression; the Potential Problem Gambler group (PPG) reported an increase that was significant when applying an alpha of 0.05, but not at the adjusted alpha level. However, the PPG group reported significantly higher baseline levels of depression pre-lockdown, and significantly higher during-lockdown depression scores. This finding is consistent with previous research that shows higher levels of depression in gamblers (12–15). Furthermore, although gamblers were more depressed both pre- and during- lockdown, and all groups increased depression scores, the change scores, (i.e., the pre- to during-lockdown increases) did not differ between groups, indicating that the increase in depression was relatively uniform across the sample, and did not differ in magnitude between gambler groups.

### Anxiety

Across the whole sample, anxiety increased significantly between pre- and during-lockdown. When examining between gambler groups, all groups reported increases in anxiety, however only the NPG group reported a significant increase. As with the depression scores, the PPG group reported higher anxiety scores at both baseline (pre-lockdown), and during lockdown than other groups, supporting previous research indicating higher levels of anxiety in gamblers (19, 20). However, although the PPG group reported higher levels of anxiety and both pre- and during-lockdown, and the NPG group reported the only significant increase, the magnitude of change in anxiety did not differ between gambler groups.

### Stress

Results indicate that across the whole sample, stress increased between pre- and during-lockdown. Within gambler groups, all groups reported increased stress levels, however only the increases in the NG group and the NPG reached significance. Although the only group not demonstrating a significant increase in stress, the PPG group nonetheless reported higher stress scores than the NG group at both pre- and during lockdown, and higher stress scores pre-lockdown that were significant, and higher stress scores that were not significantly different during-lockdown than the NPG group. This result is in accordance with previous research that found increased stress is related to gambling (22–25). The magnitude of the pre- and during-lockdown change between did not differ between groups.

#### Behavioral and Treatment Implications

Recently published research has given some indication of changes in gambling patterns. In Sweden, one study reported that higher levels of reported gambling problems were associated with a specific type of betting (sports betting) despite a decrease in sports betting availability (40). However, caution should be exercised when comparing Sweden to the UK due to the differences in both gambling legislation, and the reaction to the COVID-19 pandemic of the respective governments.

In the UK, figures from the Gambling Commission indicate that past 4-week gambling participation remained relatively stable in the initial stages of lockdown. However, mental health had been negatively affected, with up to 25% of respondents indicating their mental health had been negatively impacted (41). In relation to the current study, it is clear that lockdown has had a negative impact on the mental health of all participants in this study, not only the potential problem gambler group. However, this is particularly concerning for the gamblers in the study, who were already experiencing significantly higher levels of depression, stress, and anxiety, which appear to have been exacerbated by lockdown. Despite experiencing often severe levels of harm as a consequence of gambling, very few gamblers seek treatment for gambling disorder; in a recent review of treatment services for gambling in the UK, it was estimated that only 3% of disordered gamblers seek treatment (42). However, whilst not seeking treatment for the underlying disorder, gamblers do access healthcare more frequently that non-gamblers; previous research indicates that gamblers are twice as likely to consult a GP, five times more likely to be admitted as hospital inpatients, and eight times more likely to have received psychological counseling than non-gamblers (43).

It is possible that the increase in depression and anxiety in gamblers and non-gamblers could result in an increase in demand for mental health services, at a time where many face-to-face services are not available. As such, increased demand may be placed on online or telephone-based support services. Whilst reports suggest that demand for online gambling support services is increasing, future research will need to assess whether those experiencing gambling problems in lockdown are seeking help for the primary gambling disorder, or whether concurrent increases in depression and anxiety are reflected in increased demand for general mental health support. Future research can also identify if any observed increase in prescribing anti-depressant medication is related to gambling in lockdown.

## Limitations

Whilst providing an important cross-sectional snapshot of the immediate influence of COVID-19 and lockdown on depression, anxiety, and stress in gamblers and non-gamblers in the UK, the study was not without limitations. The screening tool used to measure the prevalence of potential gambling problems was selected due to a combination of strong psychometric properties, and brevity. However, the BPGS is not widely used, and therefore any prevalence rates measured are difficult to put in to a national and international context. Future studies could use the Problem Gambling Severity Index [PGSI, (44)] to allow classification of gambling problems on a scale of harm, and comparison with both UK and international prevalence rates. The nine-item PGSI is only four items longer than the five-item BPGS, so would not significantly increase participant burden. Furthermore, it is acknowledged that our sample may not be representative of the UK population as a whole, or of the population of those who gamble. Additionally, the sample in the current study was heavily weighted to toward female respondents; it is therefore unknown if our findings are generalisable to the general gambling population, or whether the results are more indicative of challenges faced by female gamblers.

## Conclusions

The global COVID-19 pandemic and the subsequent Government response have created an unprecedented set of circumstances for the UK public. Several factors resulting from enforced lockdown are conducive to the development, maintenance, or relapse into gambling problems. This study sought to explore the initial change is depression, anxiety, and stress in gamblers and non-gamblers in the UK, in the first weeks of lockdown. Results indicate that depression, stress, and anxiety are increasing regardless of gambler status; however, the mere fact that increases are general across all groups, should not detract from the elevated levels of depression, stress, and anxiety experienced by those experiencing gambling harm. This study provides a foundation for assessing and measuring the continuing and longer-term impacts of COVID-19 on longer term depression, anxiety, and stress in gamblers in the UK.

## Data Availability Statement

The raw data supporting the conclusions of this article will be made available by the authors, without undue reservation.

## Ethics Statement

The studies involving human participants were reviewed and approved by the University Research Ethics Committee, University of East London. The patients/participants provided their written informed consent to participate in this study.

## Author Contributions

SS was responsible for questionnaire design, data collection, and manuscript preparation. AR, HB-J, and JS were responsible for questionnaire design and manuscript preparation. All authors contributed to the article and approved the submitted version.

## Conflict of Interest

In the last 3 years, SS has received funding from the Society for the Study of Addiction (SSA), and the NIHR. He is currently employed at the NAC, part of the NIHR Biomedical Research Centre and declares no conflicts. AR has received funding from Santander, Public Health for Lincoln, The Royal Society, The Maurice and Jacqueline Bennett Charitable Trust, East Midlands RDS and internal University of Lincoln awards. She has no conflicts of interest. HB-J is the Director of The National Problem Gambling Clinic which receives funds from the National Health Service and GambleAware. She is Honorary Professor at University College London. Board member, International Society of Addiction Medicine, Board member of the International Society for the Study of Behavioural Addictions. President Elect of the Royal Society of Medicine Psychiatry Section. JS is a researcher and clinician who has worked with a range of governmental and non-governmental organizations, and with pharmaceutical and technology companies to seek to identify new or improved treatments from whom his employer (King's College London) has received honoraria, travel costs, and/or consultancy payments, but these do not have a relationship to the study and findings reported here. For a fuller account, see JS's web-page at: http://www.kcl.ac.uk/ioppn/depts/addictions/people/hod.aspx. JS is a National Institute for Health Research (NIHR) Senior Investigator and is supported by the NIHR Biomedical Research Centre for Mental Health at South London and Maudsley NHS Foundation Trust and King's College London.
